# HLA Diversity of Over 500,000 Individuals on the DATRI Register Across 18 Regions in India

**DOI:** 10.1111/tan.70908

**Published:** 2026-09-17

**Authors:** Michaela Agapiou, Nezih Cereb, Claudia Rutt, Steven G. E. Marsh, James Robinson

**Affiliations:** ^1^ Anthony Nolan Research Institute London UK; ^2^ UCL Cancer Institute London UK; ^3^ DATRI Blood Stem Cell Donors Registry, Chennai, India; ^4^ Histogenetics Ossining New York USA

**Keywords:** allele, diversity, genotype, haplotype, HLA, India

## Abstract

Registries of unrelated stem cell donors can use HLA haplotype frequencies to aid in registry management. DATRI is India's largest registry with over 550,000 donors, with donors in 35 of India's 36 states and union territories. A geographical based analysis on HLA haplotype frequencies has not previously been carried out with an Indian dataset of this size. To balance granularity and accuracy in estimating 5‐locus haplotype frequencies for HLA‐A, ‐C, ‐B, ‐DRB1 and ‐DQB1, we grouped donors into 18 regions with a minimum population size of 2000 donors. We identified the most frequent haplotype as A*33:03‐ARD~C*07:01‐ARD~B*44:03‐ARD~DRB1*07:01‐ARD~DQB1*02:01‐ARD in Odisha (6.09%), which was also the most frequent haplotype in nine other regions. Assigning the most likely diplotypes to donors, there were a total of 57,959 haplotypes and just 202 (0.3%) of those were present in all 18 regions. HLA‐B had the greatest number of alleles across the whole dataset (559) and HLA‐DQB1 the least (167). However, HLA‐C displayed the most inter‐region diversity with only 6.7% of HLA‐C alleles present in all regions (average 9.5%). We clustered the regions, using Nei's standard genetic distance, and these correlated with geography; however, three regions (North India, Kerala, and the Gujarat area) did not fit into any cluster. These regions each had one or more distinctive haplotypes that were very frequent (≥ 0.5%) in that region alone. Additionally, the Gujarat area had the lowest diversity metrics. These data can be used to inform the management of registries and contribute to our understanding of HLA diversity globally.

## 1 Introduction

Haematopoietic cell transplantation (HCT) is a potentially curative treatment for patients with haematological disorders and requires HLA compatibility between patients and donors, with the success of transplantation affected by the degree of matching [1, 2, 3]. The HLA genes reside within the human major histocompatibility complex (MHC) and are the most polymorphic genes known in the human genome [4, 5], they are crucial for immune system functions. There are six classical genes that are commonly typed and matched for in patient‐donor matching: three Class I genes (HLA‐A, ‐C, and ‐B) and three Class II genes (HLA‐DRB1, ‐DQB1, and ‐DPB1). These genes contribute to the majority of the known polymorphism in the MHC; there are 41,560 known HLA alleles in the IPD‐IMGT/HLA Database [5] (version 3.60.0, April 2025), of these 28,683 are alleles from across the three classical Class I genes and 12,877 are from the classical Class II genes.

The majority of the variation reported to date for the classical HLA genes is within the exons that encode the antigen recognition domain (ARD)—exons 2 and 3 for HLA Class I genes and exon 2 for HLA Class II. The ARDs bind self and non‐self peptide antigens that the HLA proteins present on cell surfaces to T cells (HLA Class I and Class II) and natural killer cells (primarily HLA Class I). The specific evolutionary mechanisms that have driven this diversity while maintaining allelic lineages within human populations, and even across species, are an active area of research [6]. The full extent of variation with the HLA genes remains unknown, with some published models predicting there to be 2.7–3.5 million alleles for each of the HLA Class I genes and 0.7–1.7 million alleles for HLA Class II genes in the current human population [4, 7, 8]. These figures are also likely to be an underestimate as they are focussed on ARD variation.

The diversity of HLA molecules makes finding suitably matched donors for patients in need of HCT incredibly challenging. The high degree of variation is a challenge for all demographics but particularly so for populations that are less well studied. There have been multiple efforts to both develop and validate haplotype frequency analysis [9, 10], as well as concerted efforts to characterise and record allele and haplotype frequencies from different populations [11, 12, 13]. This can be seen in resources that focus on recording frequencies, such as the Allele Frequency Net Database (AFND) [13] and HLA‐net, and those that classify alleles based on their frequencies. The HLA community has developed several catalogues of alleles from one or more data sources that categorise alleles as common or well‐documented, and some catalogues include an additional intermediate category [14, 15, 16, 17, 18, 19]. More recently, allele frequencies from 201 to 423 populations (depending on the gene) from across nine different global regions have been used to classify alleles for the six classical HLA genes into very frequent, frequent, and infrequent while intersecting this data with whether they are universally (very) frequent or locally (very) frequent [12]. These endeavours have highlighted how sensitive the calculated frequencies are to sample size, but they are able to show there are both universally frequent alleles and other alleles with distinct geographical patterns. At the haplotype level, studies from donor registries and other datasets with large and/or globally diverse populations have demonstrated similar patterns where there are some broadly frequent haplotypes and some that are geographically distinct [11, 20, 21, 22, 23].

Due to the global distribution of HLA alleles and the linkage disequilibrium between HLA genes creating haplotype blocks [24], many HCT patients with suitably matched donor(s) are likely to find them in the same region(s) that their recent ancestors are from, if established donor registries exist in those countries. However, 47% of donations are international [16] and human migration continues to spread genetic variation globally, so domestic donations will not always be an option. HLA haplotype frequencies aid in the understanding of the global distribution of HLA and are used in donor search algorithms [25, 26]. Haplotype frequencies are also used to inform registry maintenance and donor recruitment strategies as they can be the basis for modelling populations and used in calculating match likelihoods [27, 28]. The estimation of HLA haplotype frequencies is often focussed on 5‐locus haplotypes (HLA‐A, ‐C, ‐B, ‐DRB1, and ‐DQB1). The sixth gene that is also commonly used in HLA matching, HLA‐DPB1, is separated from the other five genes by a recombination hotspot, making the association between alleles of HLA‐DPB1 and other HLA genes less consistent and therefore less predictable. This increases the challenges in matching patient and donors for HLA‐DPB1 alleles and in accurately estimating haplotype frequencies that extend to this gene.

In 2023, there were ~44 million potential volunteer HCT donors in the World Marrow Donor Association (WMDA) database, an estimated ~45% of whom had been typed for all six classical HLA genes [29, 30]. While there are donors from across the globe, they are not representative of the global population; 65.6% reside in Europe and North America which is only 14.1% of the global population [29, 30, 31]. India is the most populous country in the world and has the largest global diaspora with the most emigrants in the world [32], yet there are few large‐scale studies on the HLA frequencies within India. There are five recognised registries in India, the largest is the DATRI Blood Stem Cell Donors Registry (hereby referred to as DATRI, meaning ‘donor’ in Sanskrit). DATRI was set up in 2009 and is now the largest registry in India for potential volunteer donors for HCT with over 560,000 individuals. A study in 2014 used data from DATRI's register alongside nine other Indian based data sources to calculate 5‐locus HLA haplotype frequencies based on ~18 K individuals across 14 regions, which was the largest published study on HLA frequencies in India at the time (referred to as the ‘IND10’ dataset) [33]. There are also 5‐locus haplotype frequencies deposited in AFND from an umbilical cord blood bank in India (referred to as the ‘UCBB’ dataset), for six different regions (North, East, South, West, Central and Northeast) totalling 30,027 samples. Most recently, the DKMS Foundation India (referred as the ‘DKMS India’ dataset) registry published an analysis of the HLA haplotypes on their register from over 130,000 donors, and specifically examined eight populations based on language and the state of origin of both donors' parents [34]. Studies from registries in other countries with large Indian or South Asian diaspora populations show that there is often a larger amount of HLA diversity in these populations than European ones [20, 21, 35], as is consistent with the rest of the human genome [36, 37]. Here, we present the first in‐depth analysis of the HLA profiles on the DATRI register alone, using the largest cohort of individuals based in India to date.

## 2 Methods

### 2.1 Study Population

Data from the DATRI register was shared on 29 January 2024, with some HLA genotype data updated for donors on 10 April 2024. All individuals recruited to the DATRI registry have given informed consent for their data to be used in related research. The study provides no personally identifiable data as part of the analysis outputs, and all analysis by the Anthony Nolan team has been performed on pseudo‐anonymised data. We used the genotypes for HLA‐A, ‐B, ‐C, ‐DRB1 and ‐DQB1 for this analysis, which were genotyped using sequence‐based typing methods with sequencing platforms from Illumina (San Diego, California) and Pacific Biosciences (Menlo Park, California). We removed individuals who had any data missing for HLA‐A or HLA‐B (1% of the data). Additionally, we used demographic data including date of birth, gender, country of residence and state/union territory of residence. The data for state/union territory were cleaned to place all individuals that had India as a country to have one of the official 36 states/union territories or ‘Unknown’. For example, ‘Dadra and Nagar Haveli and Daman and Diu’ is a single union territory that came from the merging of two former union territories (‘Dadra and Nagar Haveli’ and ‘Daman and Diu’) in 2020, so individuals that had one of these former names would be replaced with the current, full name.

We note that this is not an anthropological study, and that the recruitment of donors to a register is not random. We present the analysis based on a large registry dataset, as has been done by other large registries. These studies have shown that large datasets based on donor recruitment can be used as representative datasets [20, 21, 22, 23, 35].

### 2.2 Defining Geographical Regions

**FIGURE 1:**
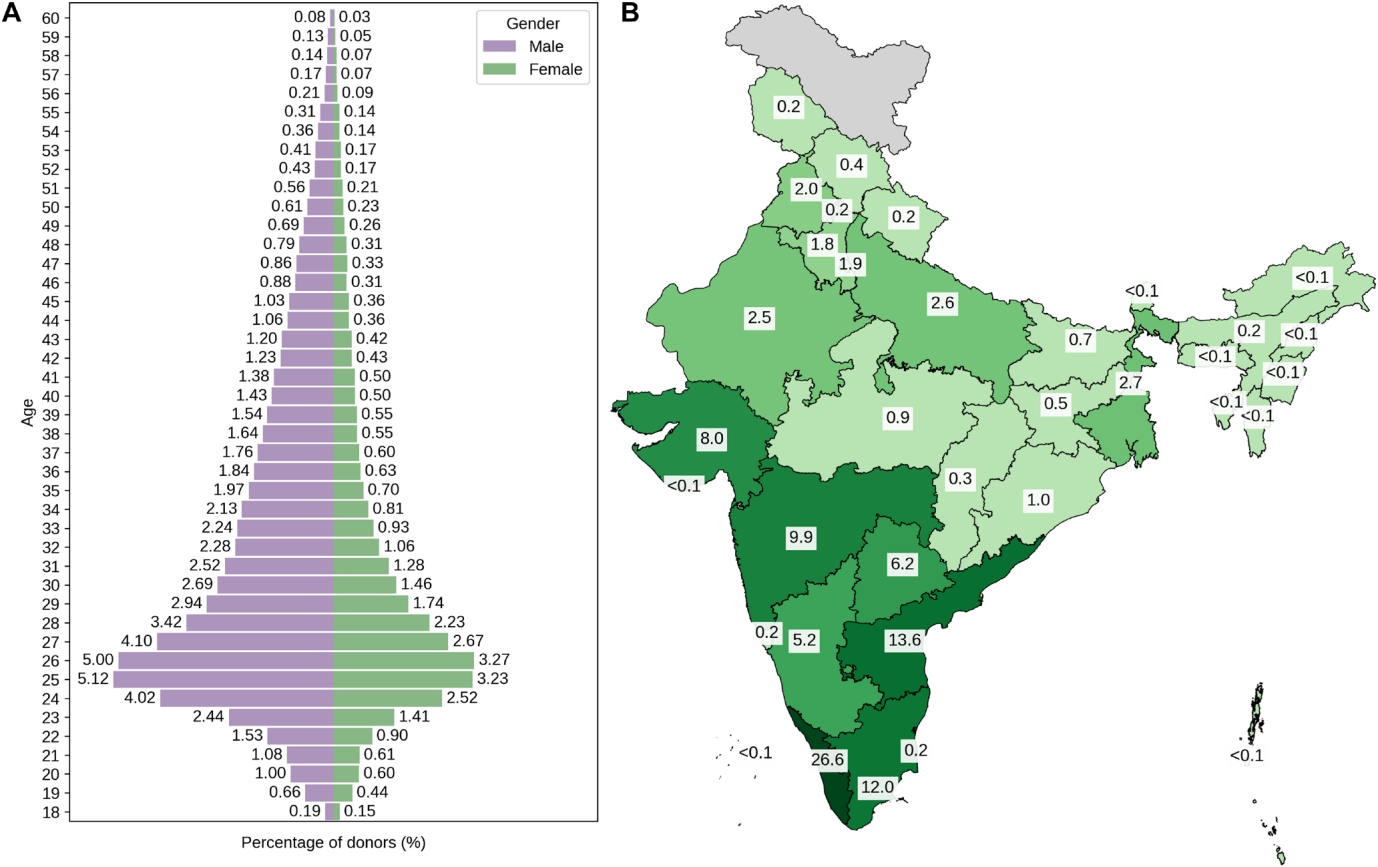
(A) Donors on DATRI register split by age and gender. (B) Map of India and the percentage of donors across the 36 states and union territories (grey: No donors).

To determine the minimum population size for a region for our downstream analysis we selected five states that had at least 10,000 individuals and compared the diversity metrics from modelled populations based on 1000, 2000, 4000 and 10,000 individuals with 100 iterations. To balance accurately capturing the diversity of the populations and the granularity between regions we chose 2000 donors as the minimum population size for a region. Those regions with less than 2000 donors were merged with neighbouring states and union territories (Table 1). Given Lakshadweep, and Andaman and Nicobar Islands are island‐based union territories these were not merged with other regions and the ~200 donors (Figure 1B) from these regions were excluded from our analysis. In total 18 regions were defined.

**TABLE 1:** Details of 18 regions formed by requiring a minimum of 2000 donors in each region.

Region	States/Union territories	Percentage of DATRI register	Percentage of region
Andhra Pradesh	Andhra Pradesh	13.59	100
Bihar	Bihar	0.72	100
Delhi	Delhi	1.89	100
Gujarat & DNHDD	Gujarat	8.01	99.47
	Dadra and Nagar Haveli and Daman and Diu	0.04	0.53
Haryana & Chandigarh	Haryana	1.8	90.58
	Chandigarh	0.19	9.42
Jharkhand & Chhattisgarh	Jharkhand	0.51	62.4
	Chhattisgarh	0.3	37.6
Karnataka	Karnataka	5.21	100
Kerala	Kerala	26.61	100
Madhya Pradesh	Madhya Pradesh	0.85	100
Maharashtra & Goa	Maharashtra	9.88	98.44
	Goa	0.16	1.56
NE India	Arunachal Pradesh	0.01	0.48
	Assam	0.18	5.86
	Manipur	0.04	1.29
	Meghalaya	0.02	0.59
	Mizoram	0.01	0.19
	Nagaland	0.01	0.23
	Sikkim	0.02	0.55
	Tripura	0.03	0.85
	West Bengal	2.72	89.96
North India	Himachal Pradesh	0.37	43.8
	Jammu and Kashmir	0.23	27.25
	Uttarakhand	0.25	28.95
Odisha	Odisha	0.95	100
Punjab	Punjab	1.99	100
Rajasthan	Rajasthan	2.5	100
Tamil Nadu & Puducherry	Tamil Nadu	11.98	98.66
	Puducherry	0.16	1.34
Telangana	Telangana	6.17	100
Uttar Pradesh	Uttar Pradesh	2.56	100

### 2.3 Haplotype and Allele Frequency Estimation

HLA genotypes for individuals on the DATRI register are mixed‐resolution data, ranging from allele groups (1 field) to P and G groups to allelic (2–4 field). Individuals who were missing data at HLA‐A or HLA‐B were excluded from the subsequent analysis. Prior to 5‐locus haplotype frequency estimation, HLA genotype strings were standardised by converting the original genotypes to all the potential ARD‐level equivalent allele names that could be represented by the original string. Multiple different proteins are covered by the same ARD group designation; we have added ‘‐ARD’ to the end of the lowest numbered allele names to reflect this. This encoding is similar but differs from P groups as null alleles that have the same or synonymous nucleotide sequences in the relevant exons as alleles that are expressed are also included in these groupings. All alleles included in each grouping can be found in Supporting Information S1. We used the latest optimisations of the Anthony Nolan software, Cactus [38, 39], which uses an EM approach [40, 41], to calculate 5‐locus haplotype frequencies and subsequently used these for allele frequencies, as previously described [20]. Haplotypes and allele frequencies, with a cutoff of 1/2*n* (where *n* is the region sample size), for each region are reported on separate sheets in the Excel file provided as Supporting Information S2.

### 2.4 Statistics and Metrics

To test for deviation from Hardy–Weinberg we performed a nested likelihood ratio test using the maximum log likelihood values produced from estimating allele frequencies using a local version of the GENE[RATE] tools, specifically the CGC (version 2.3) and CGCI (version 1.3) components [10, 42]. For this method to apply to registry data we added an uncertainty term for homozygous typing and removed alleles that are exclusively observed in ambiguous typing strings which are likely to be typing and/or data processing artefacts. These allele frequencies were highly concordant with the allele frequencies estimated by Cactus, with a mean Pearson rank correlation coefficient of 0.995.

To assess linkage disequilibrium (LD) two‐locus haplotype frequencies were estimated using GA2L (version 1.6) from the GENE[RATE] toolset. Hedrick's *D′* was calculated for overall LD between the pairs of HLA loci [43], and the standardised residuals from the GA2L output were used to evaluate the LD between pairs of alleles, a threshold of ~1.96 (akin to a *p*‐value of 0.05) was used as the threshold for designating an allele pair as being in LD, and subsequently used in the evaluation of the 5‐locus haplotypes from Cactus.

Pairwise genetic distances between regions were calculated using Nei's standard genetic distance [44]. Pairwise geographic distances were calculated using the great‐circle distance between the centroid of each region; the distances were calculated with GeoPy's great circle function, and the centroid was calculated using GeoPandas centroid function [45]. Genetic distance and geographic distance were correlated using the Mantel test, using scikit‐bio [46].

To create regional diversity metrics, individuals were assigned their most likely diplotype (pair of unambiguous ARD‐level 5‐locus haplotypes) using the haplotype frequency outputs from Cactus that match their original genotyping data. From this, subsamples were generated by randomly sampling the diplotypes of 3000 individuals from each region using Pandas sample function [47, 48]. Two metrics were calculated at both the haplotype and genotype level: percentage of distinct types and percentage of singleton types. Distinct types are the number of observed genotypes or haplotypes divided by *n* or 2*n*, respectively. Singleton types are genotypes or haplotypes that are observed in only one individual in the sampled cohort, and are also divided by *n* or 2*n*, respectively.

### 2.5 Data Visualisation and Clustering

All bar charts were made using Pandas and Matplotlib [49, 50]. Geographical visualisations were made using GeoPandas. Clustered heatmaps and dendrograms were created using seaborn [51] and the UPGMA clustering method [52]. Violin plots were also made with seaborn.

## 3 Results

### 3.1 DATRI Demographics and Regions

As of January 2024, there were nearly 540,000 donors on the DATRI register. The majority of donors are male (66%), and the mode age is 25 for men and 26 for women (Figure 1A). The majority of DATRI's donors (99%) were registered as living in India and there were donors across 35 of the 36 states and union territories of India, the exception being the northernmost union territory of Ladakh (Figure 1B). To carry out a geographical based analysis, as this is a factor often used for designing recruitment strategies, we grouped the donors from these areas into 18 regions that ranged in population size from ~3,900 to ~142,000 individuals (Table 1; Methods 2.2). Of the donors resident in India, just over half reside in the three southern states of India: Kerala, Andhra Pradesh and Tamil Nadu (Figure 1; Table 1).

### 3.2 Hardy–Weinberg Equilibrium and Overall Linkage Disequilibrium

Each locus was tested for significant deviation from Hardy–Weinberg equilibrium in each region and found no deviation. We calculated Hedrick's *D′* as a measure for overall LD between two loci, which was consistent between each region, and showed the established patterns of strong linkage between HLA‐C and HLA‐B, as well as between HLA‐DRB1 and HLA‐DQB1 (Table 2; Supporting Information S3).

**TABLE 2:** Pairwise overall linkage disequilibrium between the 5 HLA loci from the mean values of the 18 regions and ranges in parentheses.

	HLA‐A	HLA‐C	HLA‐B	HLA‐DRB1
HLA‐C	0.407 (0.370–0.459)			
HLA‐B	0.439 (0.412–0.473)	0.897 (0.886–0.914)		
HLA‐DRB1	0.287 (0.258–0.321)	0.446 (0.427–0.464)	0.531 (0.500–0.569)	
HLA‐DQB1	0.263 (0.246–0.282)	0.385 (0.371–0.403)	0.479 (0.456–0.517)	0.943 (0.909–0.962)

### 3.3 Allele, Haplotype and Genotype Frequencies

The HLA genotyping on the DATRI register is at a high‐resolution level with 98.7% of donors having data for HLA‐A, ‐B, ‐C, ‐DRB1 and ‐DQB1, and 89.2% of donors having 5‐locus genotypes that are unambiguous at the antigen recognition domain level (ARD). We were able to include 98.9% of the donors for the haplotype frequency analysis.

#### 3.3.1 Haplotype Frequencies

**FIGURE 2:**
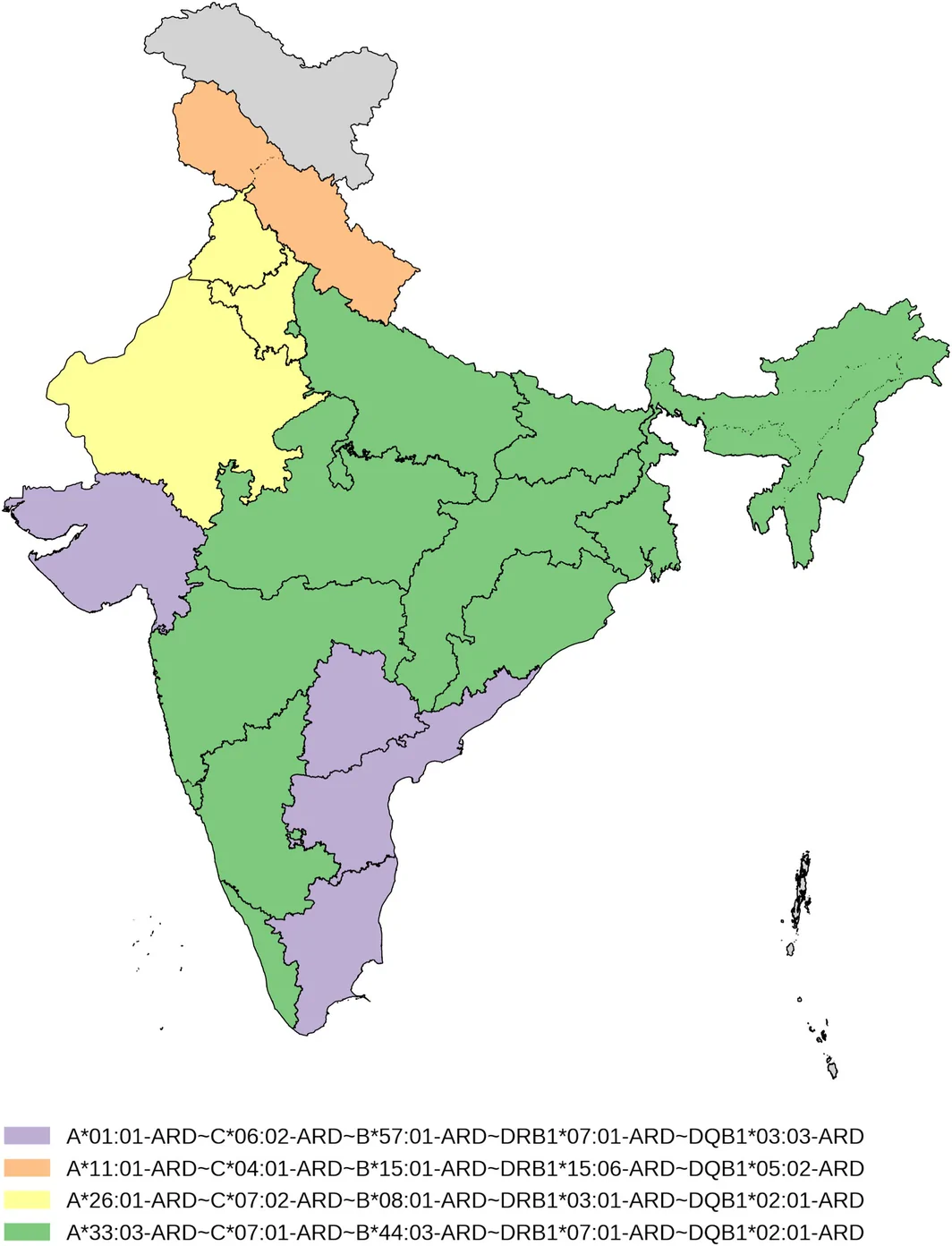
Map of India with the 18 regions coded by their most frequent haplotype.

**FIGURE 3:**
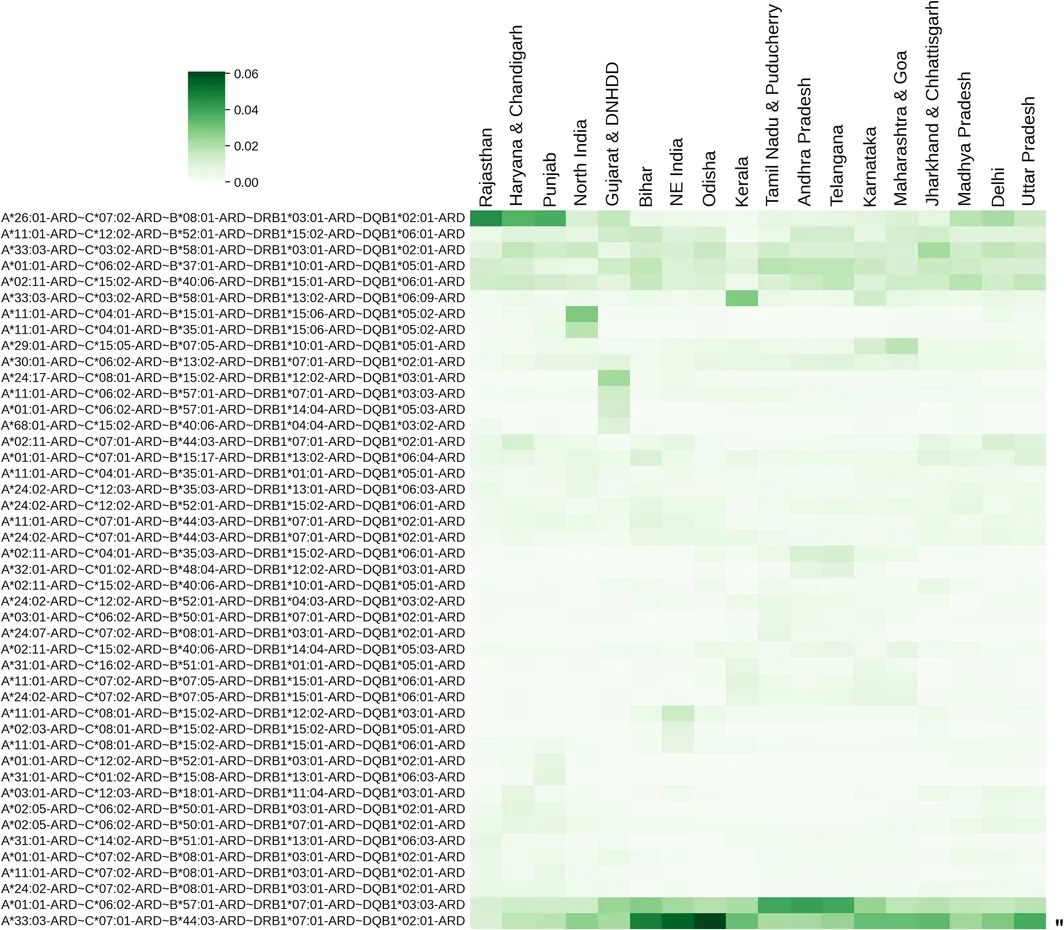
Heatmap of haplotype frequencies for the 45 haplotypes that are in the ten most frequent haplotypes for at least one region.

We estimated the 5‐locus haplotype frequencies at the ARD level for each of the 18 regions, and evaluated whether all pairwise combinations of alleles that comprise each haplotype are in LD (Supporting Information S2). The most common frequently occurring haplotype was A*33:03‐ARD~C*07:01‐ARD~B*44:03‐ARD~DRB1*07:01‐ARD~DQB1*02:01‐ARD, which was the most frequent haplotype in ten regions (Figure 2—green). Combining the 10 most frequent haplotypes from each region results in 45 distinct haplotypes, once duplicates are removed (Figure 3). The A*33:03‐ARD~C*07:01‐ARD~B*44:03‐ARD~DRB1*07:01‐ARD~DQB1*02:01‐ARD haplotype was one of only two haplotypes that were in every region's ten most frequent haplotype list, ranging from 1.05% in Rajasthan to 6.09% in Odisha. This is very similar to the IND10 dataset where this haplotype was in the ten most frequent haplotypes for all fourteen regions and highest in Odisha at 7.7%. In the UCBB and DKMS India datasets, neither of which specified Odisha as a region, this was one of the top three most frequent haplotypes in all of their regions. The other haplotype we observed in every region's ten most frequent haplotype was A*01:01‐ARD~C*06:02‐ARD~B*57:01‐ARD~DRB1*07:01‐ARD~DQB1*03:03‐ARD, which ranged in frequency from 1.14% in Rajasthan to 4.05% in Andhra Pradesh and was also the most frequent haplotype for four regions (Figure 2—yellow). Both haplotypes had alleles that were all in LD, and the frequencies of these haplotypes and many of the other top 10 haplotype results were concordant with other datasets.

There were 18 haplotypes (out of a possible 63) that were regionally distinct, in that they were frequent (≥ 0.05%) in just one region but not even half as frequent in any other region. Two of the most striking examples of these were: A*11:01‐ARD~C*04:01‐ARD~B*15:01‐ARD~DRB1*15:06‐ARD~DQB1*05:02‐ARD, present in the North India region at 2.87% but had a mean frequency of 0.15% across the other 17 regions, and A*24:17‐ARD~C*08:01‐ARD~B*15:02‐ARD~DRB1*12:02‐ARD~DQB1*03:01‐ARD in the Gujarat & DNHDD region at 2.28% but had a mean frequency of 0.13% for the other regions. Both haplotypes had alleles that were all in LD with each other. Additionally, the A*11:01‐ARD~C*04:01‐ARD~B*15:01‐ARD~DRB1*15:06‐ARD~DQB1*05:02‐ARD haplotype has not been seen in any other datasets at a frequency as high as 2.87%, although few studies have included the North India region. Where comparisons could be made, the other regionally distinct haplotypes were consistent with other datasets.

#### 3.3.2 Genetic Distance

**FIGURE 4:**
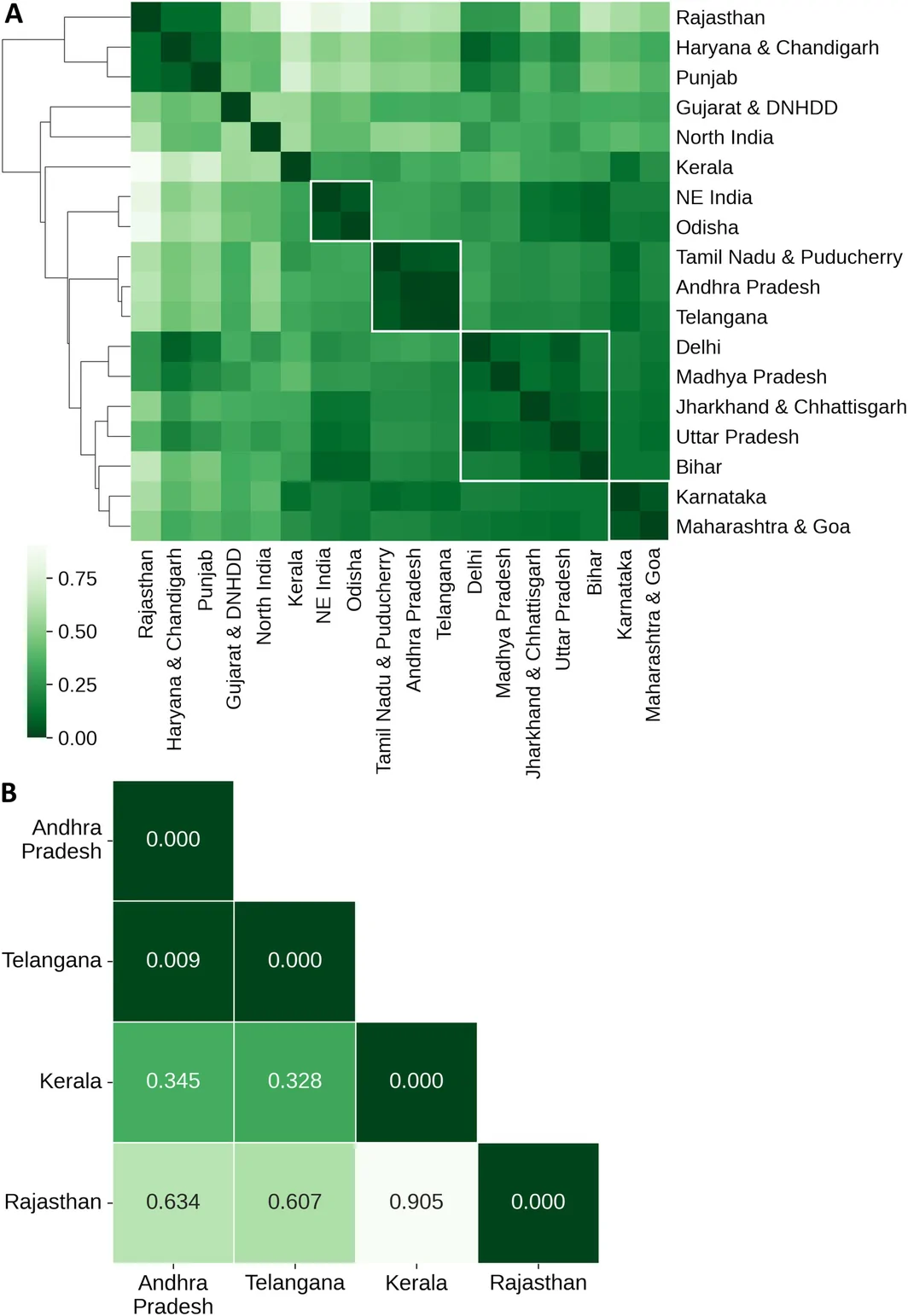
(A) Heatmap of the pairwise Nei's standard genetic distance between 18 regions on the DATRI register, with an UPGMA dendrogram. White boxes highlight four clusters within a large group of 12 regions. (B) Heatmap of the pairwise Nei's standard genetic distance for four regions: Andhra Pradesh and Telangana (the closest pair), and Kerala and Rajasthan (the most distant pair).

To examine the similarity between regions on the DATRI register, we calculated Nei's standard genetic distance using the regional haplotype frequencies (Figure 4A; Supporting Information S4). There is a very distinct cluster of the three northwestern regions (Rajasthan, Punjab, and Haryana & Chandigarh; Figure 4A), all of which have the same most frequent haplotype—A*26:01‐ARD~C*07:02‐ARD~B*08:01‐ARD~DRB1*03:01‐ARD~DQB1*02:01‐ARD (Figure 2). There were three regions that appear to be outliers, in that they did not cluster next to the regions with which they had the smallest distance score from; these were: Gujarat & DNHDD, North India, and Kerala, which were closest to Madhya Pradesh, Delhi and Karnataka, respectively. Instead, these three regions are separated from the aforementioned northwestern cluster but not embedded into any of the remaining clusters. This is consistent with these three regions having one or more regionally distinct frequent haplotypes (Figure 3). The remaining 12 regions form a series of 4 clusters (Figure 4A—white boxes) which are formed of geographically proximal regions and there is a high degree of similarity between these four clusters as well as within.

Looking at the pairwise distance scores, the mean across all comparisons was 0.277. The closest pair was Andhra Pradesh and Telangana with 0.009 and the furthest pair was Rajasthan and Kerala with 0.905 (Figure 4B). Taking each region's closest distance score, the mean was 0.088; however, this was much higher for North India and Gujarat & DNHDD which were closest to Delhi (0.264) and Madhya Pradesh (0.267), respectively. The mean for the furthest scores from each region was 0.632, and Delhi had the lowest of these with a score of 0.366 to Kerala. There was a moderate correlation between the pairwise genetic distances and geographical distances between regions (*r* = 0.537, *p* = 0.001).

#### 3.3.3 Allele Frequencies

From the haplotype frequencies, we calculated the allele frequencies for HLA‐A, ‐B, ‐C, ‐DRB1, ‐DQB1 for each region (Supporting Information S2). We found all ten of the previously reported ‘universally very frequent alleles’ in every region and we found four out of the five alleles that are ‘locally very frequent’ to ‘South & West & Central Asia’ (SWA) in every region [12]. The locally very frequent allele we did not find in every region was DRB1*11:03‐ARD, which was most frequent in Gujarat & DNHDD at only 0.06%, so this allele is probably localised elsewhere in the SWA region. While DRB1*11:04‐ARD was present in all regions, its frequency was markedly higher in northern Indian regions (up to 4.4%) than southern ones (as low as 0.2%).

**FIGURE 5:**
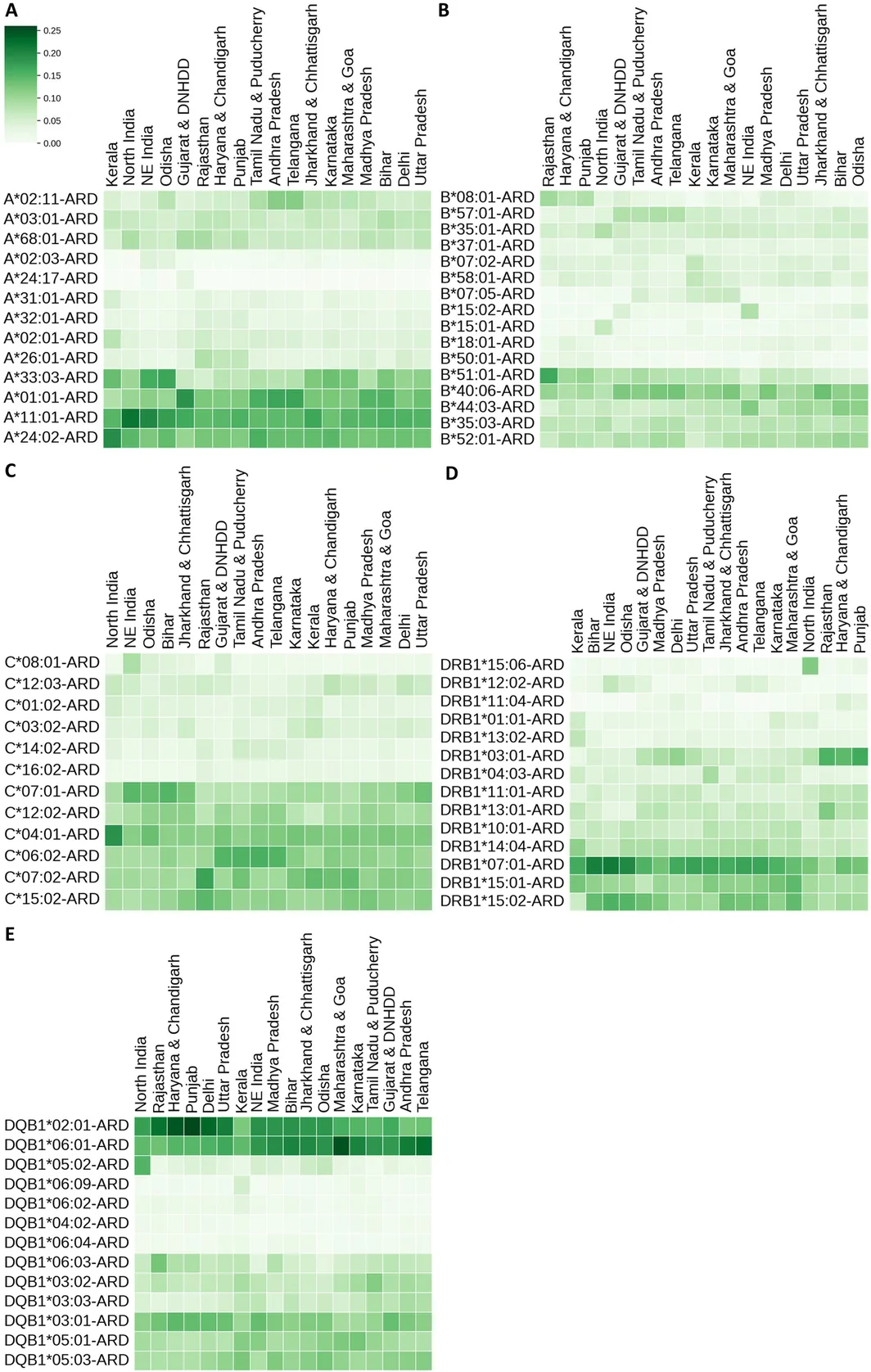
Heatmaps of allele frequencies for HLA‐A (A), HLA‐B (B), HLA‐C (C), HLA‐DRB1 (D), and HLA‐DQB1 (E) for alleles that are in the ten most frequent alleles for at least one region.

The most frequent HLA‐A allele was A*11:01‐ARD at 21.8% in the North India region, this was the most frequent HLA‐A allele in twelve of the eighteen regions (Figure 5A). When taking the top 10 HLA‐A alleles for all regions and removing duplicates there were 13 distinct alleles, and 9 of these were consistently in the top 10 across the eighteen regions. Despite being part of the most frequent haplotype, A*33:03‐ARD was not the most frequent HLA‐A allele in any region but it was in the top 10 across all regions. A*24:17‐ARD was the only allele in the top 10 for just a single region, the Gujarat & DNHDD region at 3.4%, which had a mean frequency of 0.28% in the other regions.

The most frequent HLA‐B allele was B*51:01‐ARD at 16.5% in Rajasthan, which was the most common HLA‐B allele in the neighbouring regions of Punjab and Haryana & Chandigarh (Figure 5B). B*40:06‐ARD was the most frequent HLA‐B allele in twelve of eighteen regions. HLA‐B had the least consistency for the top 10 alleles between regions with only six alleles common to all regions, and a total of 16 alleles included across all the lists. This is in keeping with HLA‐B being the most diverse of the HLA genes, as it has the highest number of reported alleles on the IPD‐IMGT/HLA Database [5]. Consequently, we see that the HLA‐B frequency heatmap is the most muted of the five genes.

C*04:01‐ARD was the most common HLA‐C allele at 18.8% in the North India region, which was also the most common HLA‐C allele for three other regions (Figure 5C). There were 7 HLA‐C alleles that were consistently in the top 10 lists for all regions. However, unlike the other HLA genes, there was not a distinct HLA‐C allele that was the most frequent allele for more than half of the regions; C*07:01‐ARD was the top ranked HLA‐C allele in five regions, and C*07:02‐ARD and C*04:01‐ARD were each the top HLA‐C allele in four different regions.

The most frequent HLA‐DRB1 allele was DRB1*07:01‐ARD in the Northeast India region at 21.2%, and this was also the most frequent HLA‐DRB1 allele for 13 other regions (Figure 5D). Similar to HLA‐C, there were 7 alleles that were consistently in the top 10 most frequent HLA‐DRB1 allele lists across the regions. However, when comparing the HLA‐C and HLA‐DRB1 heatmaps, the top allele frequencies are much more concentrated on just three alleles for HLA‐DRB1 compared to a darker band of six alleles for HLA‐C (Figure 5C & Figure 5D). Also notable is that DRB1*15:06‐ARD is very distinct to the North India region, which is observed at a frequency of 12.0% while the mean frequency across the other regions is 1.5%.

Finally, the most frequent HLA‐DQB1 allele was DQB1*02:01‐ARD in Punjab at 25.7%, this was the most frequent allele for seven regions (Figure 5E). As we are studying alleles at the ARD level, DQB1*02:01‐ARD here represents 90 different two‐field proteins, including those produced by the DQB1*02:01:01:01 and DQB1*02:02:01:01 alleles which are both ‘Common’ alleles across all populations [18]. DQB1*02:01‐ARD is in strong linkage with DRB1*07:01‐ARD, typically this association is with the full‐length DQB1*02:02 protein, so it is not surprising to observe these two alleles as the most frequent Class II alleles together. However, DQB1*02:01‐ARD is particularly high in Punjab where the two‐locus haplotype DRB1*03:01‐ARD~DQB1*02:01‐ARD is even more frequent than DRB1*07:01‐ARD~DQB1*02:01‐ARD (16.1% and 9.5%, respectively), and is likely associated with the DQB1*02:01 full‐length protein. This was also observed in two of Punjab's neighbouring regions, Rajasthan and Haryana & Chandigarh, but not in the four other regions where DQB1*02:01‐ARD was the most frequent allele. There were 8 alleles that were consistently in the top 10 most frequent HLA‐DQB1 allele lists across the regions. Similar to HLA‐DRB1, the top allele frequencies were highly concentrated, this time on just two alleles—DQB1*02:01‐ARD and DQB1*06:01‐ARD, the latter was the most frequent allele for the other 11 regions. These alleles were consistently the top two alleles in 16 regions, and in the two regions where they were not the top two (Kerala and North India) they were two of the top three most frequent HLA‐DQB1 alleles.

### 3.4 Allele, Haplotype and Genotype Diversity

From the haplotype frequencies we assigned every donor who had been included in the haplotype frequency analysis their most likely diplotype, 97% of these assignments had a relative probability of greater than 50% (Figure S1). This allows us to examine the diversity of alleles, haplotypes and genotypes on the DATRI register.

#### 3.4.1 Allele Diversity

**FIGURE 6:**
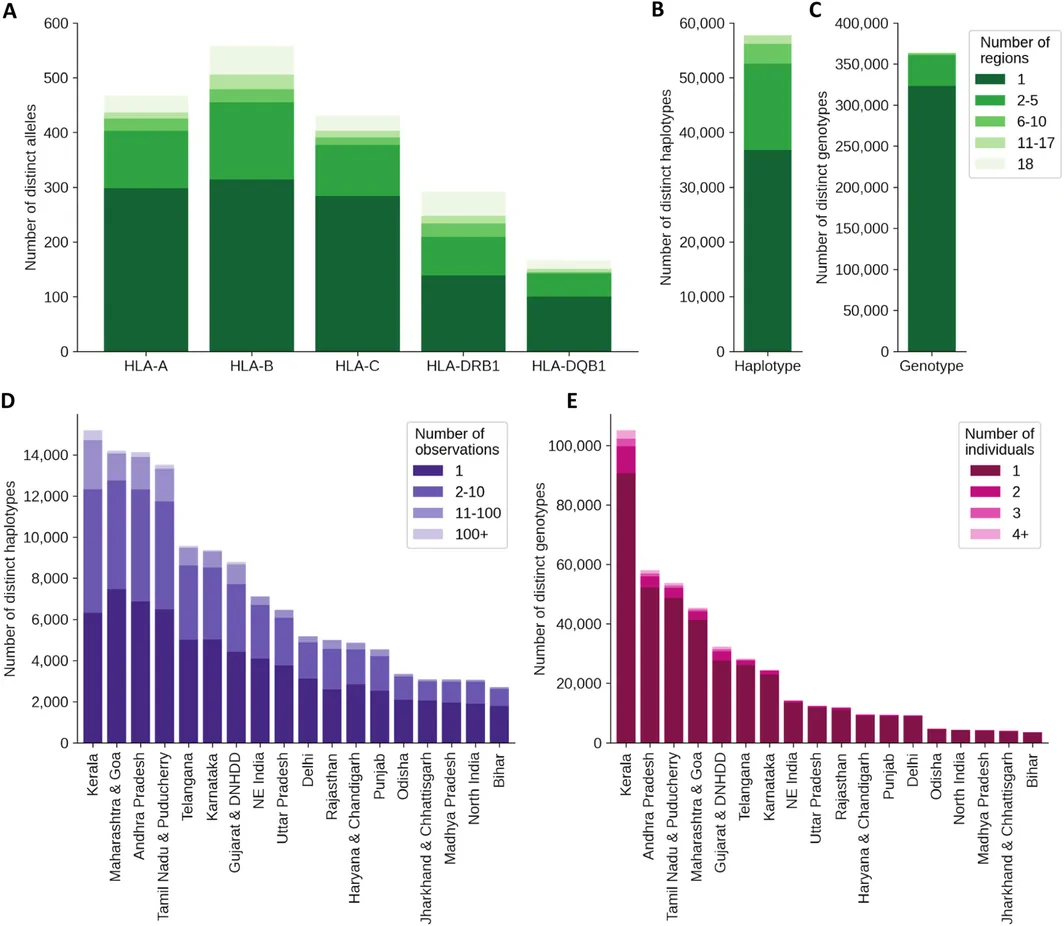
(A–C) Number of distinct alleles by gene (A), haplotypes (B), and genotypes (C) grouped into how many regions they were observed in. (D–E) Number of distinct haplotypes (D) and genotypes (E) for each region grouped by the number of individuals that are predicted to have that haplotype or genotype.

First, we looked at how many distinct alleles there were at each locus in the DATRI register and how many were present in more than one region. The average across the five loci was 384 alleles; HLA‐B had the most with 559, and HLA‐DQB1 had by far the fewest with 167 (Figure 6A). For almost all loci, more than half of the alleles (mean: 59.1%) are observed in only one region (Figure 6A—darkest green segment), for HLA‐DRB1 this is the case for 47.6% of alleles. When we remove alleles that are only observed in one individual then 41.5% of alleles are present in just one region (Figure S2A). There are small number of alleles that are present in all 18 regions (Figure 6A—lightest green segment), as a percentage HLA‐C had the least with 6.7% and HLA‐DRB1 had the greatest with 15.1%. While HLA‐B had the greatest number of alleles over the whole register, HLA‐C showed more inter‐region diversity.

#### 3.4.2 Haplotype and Genotype Diversity

From the nearly 530,000 donors there were 57,959 haplotypes; 63.5% of these were observed in just one region and only 202 (0.3%) were in all 18 regions (Figure 6B). After removing haplotypes that are present in just one individual, the number of haplotypes decreases to 27,175 and the proportion of haplotypes present in just one region is just 22.1%; however most are still only present in 2–5 regions (Figure S2B). As expected, the diversity at the genotype level is even greater; there are 363,456 genotypes from the nearly 530,000 donors, and 88.9% of these genotypes were present in a single region, while only 7 genotypes were found across all 18 regions (Figure 6C). These 7 genotypes can be described as combinations of just 5 distinct haplotypes (Table 3). After removing genotypes that are from single individuals, the number of genotypes decreases to 57,000 and the proportion of genotypes present in a single region is 29.0% (Figure S2C). The dramatic impact of removing singleton haplotypes and genotypes demonstrates the contribution of singletons to diversity on the register as a whole and between regions. However, there are still proportionally few haplotypes and genotypes that are present in six or more regions.

**TABLE 3:** The 7 genotypes observed in all 18 regions with their likely haplotypes and the haplotype frequency range observed across the 18 regions.

Unphased genotype	Haplotypes (#)	Haplotype frequency range
A*01:01‐ARD, 02:11‐ARD; C*06:02‐ARD, 15:02‐ARD; B*40:06‐ARD, 57:01‐ARD; DRB1*07:01‐ARD, 15:01‐ARD; DQB1*03:03‐ARD, 06:01‐ARD	A*01:01‐ARD~C*06:02‐ARD~B*57:01‐ARD~DRB1*07:01‐ARD~DQB1*03:03‐ARD (1)	1.14%–4.05%
	A*02:11‐ARD~C*15:02‐ARD~B*40:06‐ARD~DRB1*15:01‐ARD~DQB1*06:01‐ARD (2)	0.38%–1.85%
A*01:01‐ARD, 33:03‐ARD; C*06:02‐ARD, 07:01‐ARD; B*44:03‐ARD, 57:01‐ARD; DRB1*07:01‐ARD, 07:01‐ARD; DQB1*02:01‐ARD, 03:03‐ARD	A*01:01‐ARD~C*06:02‐ARD~B*57:01‐ARD~DRB1*07:01‐ARD~DQB1*03:03‐ARD (1)	—
	A*33:03‐ARD~C*07:01‐ARD~B*44:03‐ARD~DRB1*07:01‐ARD~DQB1*02:01‐ARD (3)	1.05%–6.09%
A*01:01‐ARD, 33:03‐ARD; C*03:02‐ARD, 06:02‐ARD; B*57:01‐ARD, 58:01‐ARD; DRB1*03:01‐ARD, 07:01‐ARD; DQB1*02:01‐ARD, 03:03‐ARD	A*01:01‐ARD~C*06:02‐ARD~B*57:01‐ARD~DRB1*07:01‐ARD~DQB1*03:03‐ARD (1)	—
	A*33:03‐ARD~C*03:02‐ARD~B*58:01‐ARD~DRB1*03:01‐ARD~DQB1*02:01‐ARD (4)	0.66%–2.24%
A*02:11‐ARD, 26:01‐ARD; C*07:02‐ARD, 15:02‐ARD; B*08:01‐ARD, 40:06‐ARD; DRB1*03:01‐ARD, 15:01‐ARD; DQB1*02:01‐ARD, 06:01‐ARD	A*02:11‐ARD~C*15:02‐ARD~B*40:06‐ARD~DRB1*15:01‐ARD~DQB1*06:01‐ARD (2)	—
	A*26:01‐ARD~C*07:02‐ARD~B*08:01‐ARD~DRB1*03:01‐ARD~DQB1*02:01‐ARD (5)	0.26%–4.5%
A*02:11‐ARD, 33:03‐ARD; C*07:01‐ARD, 15:02‐ARD; B*40:06‐ARD, 44:03‐ARD; DRB1*07:01‐ARD, 15:01‐ARD; DQB1*02:01‐ARD, 06:01‐ARD	A*02:11‐ARD~C*15:02‐ARD~B*40:06‐ARD~DRB1*15:01‐ARD~DQB1*06:01‐ARD (2)	—
	A*33:03‐ARD~C*07:01‐ARD~B*44:03‐ARD~DRB1*07:01‐ARD~DQB1*02:01‐ARD (3)	—
A*33:03‐ARD, 33:03‐ARD; C*03:02‐ARD, 07:01‐ARD; B*44:03‐ARD, 58:01‐ARD; DRB1*03:01‐ARD, 07:01‐ARD; DQB1*02:01‐ARD, 02:01‐ARD	A*33:03‐ARD~C*03:02‐ARD~B*58:01‐ARD~DRB1*03:01‐ARD~DQB1*02:01‐ARD (4)	—
	A*33:03‐ARD~C*07:01‐ARD~B*44:03‐ARD~DRB1*07:01‐ARD~DQB1*02:01‐ARD (3)	—
A*33:03‐ARD, 33:03‐ARD; C*07:01‐ARD, 07:01‐ARD; B*44:03‐ARD, 44:03‐ARD; DRB1*07:01‐ARD, 07:01‐ARD; DQB1*02:01‐ARD, 02:01‐ARD	A*33:03‐ARD~C*07:01‐ARD~B*44:03‐ARD~DRB1*07:01‐ARD~DQB1*02:01‐ARD (3)	—
	A*33:03‐ARD~C*07:01‐ARD~B*44:03‐ARD~DRB1*07:01‐ARD~DQB1*02:01‐ARD (3)	—

#### 3.4.3 Intra‐Regional Diversity

We next looked at the haplotype and genotype diversity within each region. For each haplotype in a region, we looked at whether it was observed once (darkest purple), 2–10 times, 11–100 times, 100+ times (lightest purple; Figure 6D; Supporting Information S4). On average, more than half of the haplotypes in a region are observed only once (56.3%), however, this percentage is greatly affected by the population size of a region with the larger regions having fewer haplotype singletons. Additionally, regions with more donors have a greater proportion of haplotypes that are present more than 100 times. This is a very typical pattern in HLA haplotype frequencies where there are a small number of haplotypes that are very frequent, some that are frequent, and then very many haplotypes that are rare, though the frequency thresholds for these categories varies based on the sample size. This is a phenomenon that we see for every region (Figure S3). At the genotype level, the mean percentage of genotypes observed only once in a region (i.e., in one individual) rises to 93.6% (Figure 6E; Supporting Information S4), and very few genotypes are present in 4 or more individuals in any region.

#### 3.4.4 Inter‐Regional Diversity

**FIGURE 7:**
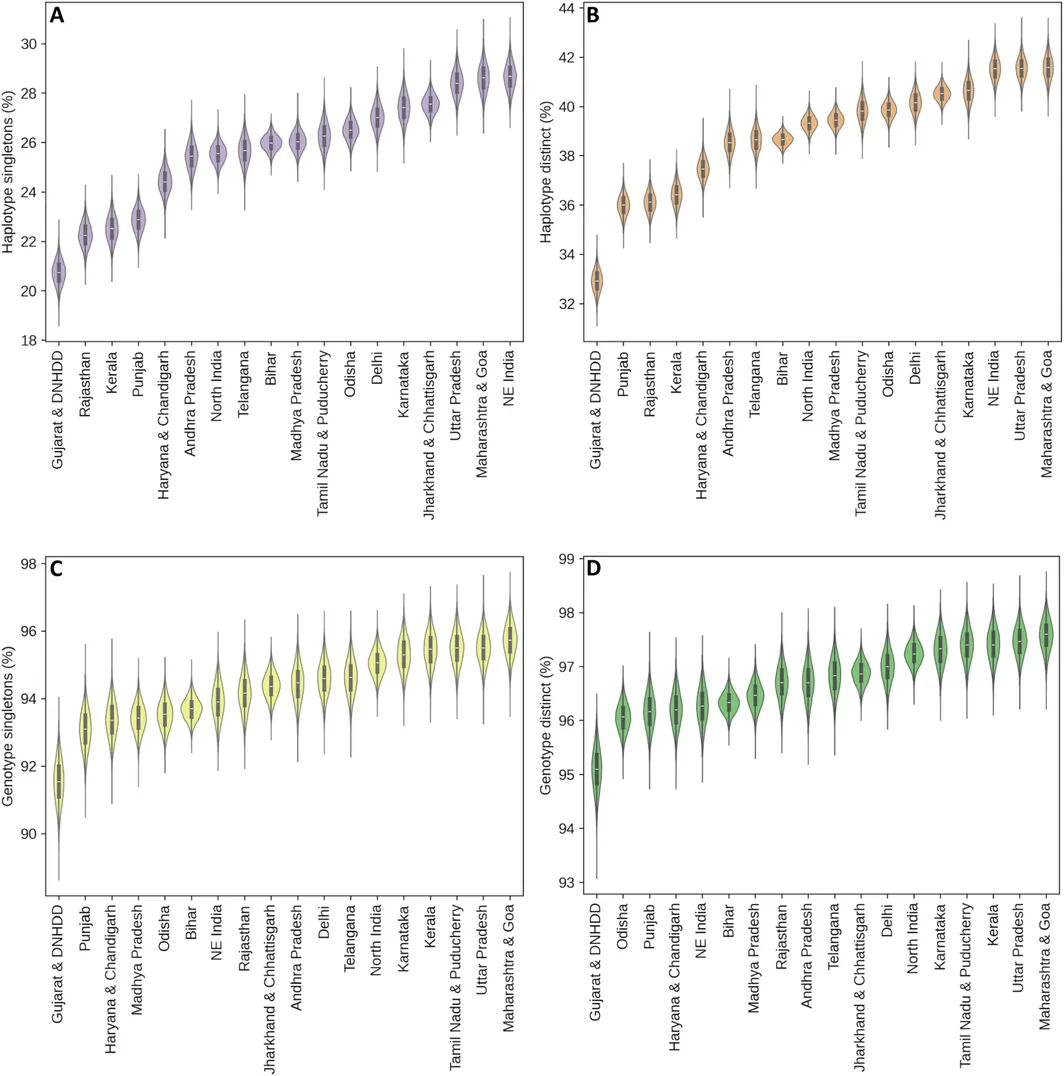
Violin plots showing the distribution of the percentages of haplotypes that are singletons (A), distinct haplotypes (B), singleton genotypes (C), and distinct genotypes (D) for each region after random subsampling of 3000 individuals from each of the regions 10,000 times.

To compare regions with large differences in population sizes, we subsampled all regions multiple times prior to calculating the percentage of distinct haplotypes and genotypes and the percentage of singleton individuals (Figure 7; Supporting Information S4). The Gujarat & DNHDD region was consistently the lowest for these metrics. While the Maharashtra & Goa region and Uttar Pradesh were in the top three across all metrics. The Northeast India region had the highest percentage of haplotype singletons (Figure 7A) but dropped to the 12th ranking in percentage of genotype singletons (Figure 7C).

## 4 Discussion

The DATRI register has over 550,000 donors from across India, the most populous country in the world with a complex history of migration and the subject of active anthropological and genomic research [53, 54, 55]. Several analyses point to there being a significant influence on the current genomic patterns in mainland India from the mixture of two to four ancestral populations, and a North–South cline [53, 55, 56, 57, 58]. While this is not an anthropological study, we present this work to understand the current regional HLA diversity on the DATRI register. We expect each region to be made of its own heterogeneous subpopulations, but we do see a correlation with geography in our data and in particular a very close similarity between three of the northwestern states (Punjab, Rajasthan and Haryana & Chandigarh) each of which is least similar to southern states (Figure 4A).

Prior to this study, the DATRI register had been included in an analysis of HLA haplotype frequencies, which covered 14 of India's states and union territories, with a minimum population size of 200 individuals [33]. Over the last decade, the DATRI register has grown considerably; thus, we have been able to cover a larger area of India with at least 10 times more depth, and in the extreme example of Kerala with 100‐fold more depth. The increased breadth of this dataset allowed us to include four additional regions in our analysis: Gujarat & DNHDD, Jharkhand & Chhattisgarh, North India, and Telangana, where the latter three regions have not been specifically reported on previously by any large study. Alongside Kerala, we found Gujarat & DNHDD and North India to be particularly distinctive regions, demonstrating the benefit of the additional granularity reported here for understanding the distribution of HLA haplotypes on the DATRI register. Furthermore, the granularity combined with the increased depth has highlighted the immense diversity of HLA types regionally. Hundreds of alleles were only present on the register in one region, even when removing those that are present in single individuals and even when considering ARD‐level alleles. Inevitably, this allelic variation drives even greater numbers of haplotypes and genotypes, to the extent that even within regions the majority of genotypes are only observed once, including in Kerala which was the largest region (~142,000 individuals).

The A*11:01‐ARD~C*04:01‐ARD~B*15:01‐ARD~DRB1*15:06‐ARD~DQB1*05:02‐ARD haplotype demonstrates the benefit of the breadth of data we have, as it has not previously been observed at the high frequency we found in North India, where it was the most frequent haplotype for this region. While this is a registry dataset and not a population study, the high frequency of this haplotype suggest that this could be a signal of a locally distinct haplotype. North India is a previously understudied region, and here each of the alleles in this haplotype were more frequent than any other region. However, B*15:01‐ARD and DRB1*15:06‐ARD were particularly localised to North India in this study; they were observed at 6.7% and 12.0%, respectively, while elsewhere they were only present at ~1% (Figure 5). Globally, B*15:01 is a universally frequent allele, but it is also locally very frequent in the Northeast Asia region, while DRB1*15:06 is a locally frequent allele in the SWA region [12]. Additionally, the third most frequent haplotype in North India, had the same basic combination of alleles except B*35:01‐ARD replaced with B*15:01‐ARD (Figure 3). While the B*15:01‐ARD variant of this haplotype was more frequent than the B*35:01‐ARD in most regions, at the allele level, B*35:01‐ARD allele was more frequent than B*15:01‐ARD in all 18 regions. Given the strong linkage between C*04:01‐ARD and B*35:01‐ARD, one might expect the B*35:01‐ARD variant of this haplotype to have arisen first and the B*15:01‐ARD haplotype variant becoming established subsequently, however we currently do not have strong evidence either way. We did not find any previous studies that evidence why these haplotypes might be particularly frequent in the North India region and we cannot attribute this to any particular group within the region, but it could reflect this region's proximity to other areas of Asia, as it borders three countries and as the most northerly region it is close to both Central Asian and East Asian countries. Additionally, the mountainous geography of this region, with a large proportion of the region being part of the Western Himalayas, may have reduced the genetic flow between this area and other regions of India, leading it to have a more distinct haplotype frequency profile.

The depth of the dataset also highlights notable but lower frequency haplotypes. One of the most well‐studied haplotypes, due to its commonality in European populations and association with different diseases, is the A1~B8~DR3 haplotype [59]. For the British/Irish North West European subpopulation on the Anthony Nolan register, we previously found, using the same software as in this study, the most common ARD‐level five locus version of this haplotype to be A*01:01‐ARD~C*07:01‐ARD~B*08:01‐ARD~DRB1*03:01‐ARD~DQB1*02:01‐ARD (also called ancestral haplotype 8.1; AH8.1) at frequency of 8.41% [20]. On the DATRI register, the most common ARD‐level haplotype for A1~B8~DR3 carries a C*07:02‐ARD instead of C*07:01‐ARD, despite C*07:01‐ARD being similarly frequent as C*07:02‐ARD across the regions (mean frequencies of 10.3% and 11.5% respectively; Figure 5C). This is consistent with a study that specifically looked at the A1~B8~DR3 haplotype in Northern India [60], the DKMS India and UCBB datasets, and studies that include the Indian diaspora [20, 21]. The A*01:01‐ARD~C*07:02‐ARD~B*08:01‐ARD~DRB1*03:01‐ARD~DQB1*02:01‐ARD haplotype (previously termed AH8.1v) is the 10th most common haplotype in Rajasthan and was observed in all regions, unlike AH8.1 which was only predicted to be at least one of the haplotypes for individuals in 8 regions. AH8.1v is also very similar to the most common haplotype in the northwestern regions, that differs at HLA‐A with A*26:01‐ARD rather than A*01:01‐ARD (previously termed AH8.2). It has been proposed that AH8.1 and AH8.1v share a common ancestral haplotype and AH8.2 may have arisen through recombination from AH8.1v [61]. It is also worth noting that the HLA‐C~HLA‐DQ stretch common to both AH8.1v and AH8.2 haplotypes, has since also been observed in large population studies carried out in Saudi Arabia [62, 63], with multiple different HLA‐A alleles being among the top 10 most frequent alleles (including A*26:01, but not A*01:01). As more individuals globally are sequenced for their HLA genes at the ARD level or higher, these data will likely contribute to our understanding how variation of ancestral haplotypes has arisen over time.

Characterising regions in India for their HLA allele and haplotype frequency, and in relation to each other, aids in patient‐donor matching analysis for HCT which can feed into donor recruitment and registry planning [25, 26, 27, 28, 33]. While matching alleles for the DNA sequence at the full length of the gene, rather than just the ARD, improves patient outcomes [1, 3], patients and donors are typically typed and matched at the ARD level due to restrictions in genotyping technologies and cost. Here, we focussed on estimating the haplotype frequencies for five of the classical HLA genes, future work into both higher resolution haplotypes and six‐locus haplotype frequencies could be worthwhile for the more common 5‐locus ARD‐level haplotypes where there might be discernible geographical patterns, especially as 86% of donors on the DATRI register with 5‐locus genotyping also have genotyping for HLA‐DPB1, which is much higher than the average across all WMDA donors. For rarer haplotypes, accuracy would be more challenging to verify, as even at the ARD‐level analysis presented here we see large numbers of singleton alleles and haplotypes, and it is likely that rare alleles and haplotype are underestimated or missing. To further enhance our understanding of HLA haplotype distributions within India, future work could be carried out using the ethno‐linguistic data held for many donors on the DATRI register. In combination with more granular geographical data (e.g., city level or rural vs. urban populations) for the states and union territories with large numbers of donors, these analyses could reveal further distinct haplotype frequency patterns.

Geography is one of the key demographic factors that donor registries can use to design their recruitment strategies. Here, we have analysed the largest dataset of HLA profiles for potential donors in India to date in a geographically informed manner. We have presented the large amount of diversity that exists within and between the 18 regions studied, and alongside other datasets, found some consistent patterns in HLA haplotype distributions that can potentially aid in registry maintenance and recruitment. We have also highlighted examples that demonstrate that data for specific regions, not just at a country‐wide level, contribute a richer understanding of the HLA haplotype frequencies and diversity on the register.

## Funding

This work was supported by National Institute for Health and Care Research, NIHR205433.

## Conflicts of Interest

The authors declare no conflicts of interest.

## Supporting information


**Supporting Information S1:** ARD group designations and the alleles present in the dataset that map to the designations. Generated using IPD‐IMGT/HLA Database version 3.57.

**Supporting Information S2:** Allele frequencies for HLA‐A, ‐B, ‐C, ‐DRB1 and ‐DQB1, 5‐locus haplotype frequencies and their LD status for each of the 18 geographical regions.

**Supporting Information: S3:** Pairwise Hedrick's *D′* values for overall LD between loci for each of the 18 geographical regions.

**Supporting Information S4:** Pairwise Nei's standard genetics distance between each of the 18 geographical regions based on 5‐locus haplotype frequencies. Inter‐region diversity and intra‐region diversity metrics.

**Figure S1:** Histogram with 100 bins of the number of individuals on the DATRI register by the relative probability of their diplotype prediction based on our EM‐based algorithm for calculating haplotype frequency.
**Figure S2:** Number of distinct alleles by gene (A), haplotypes (B), and genotypes (C) when singletons for each of these have been removed and the remaining are grouped into how many regions they were observed in.
**Figure S3:** For all 18 regions, the cumulative percentage of donors who are covered for their most likely haplotypes, with haplotypes are sorted by the haplotype frequency rank (x‐axis). Dotted lines and arrow indicate when 50% of a region's population is covered by the annotated number of haplotypes.

## Data Availability

Research data for this project is not shared. Summary data representing the frequencies of alleles and haplotypes generated as part of this study are available in the Supporting Information of this article.
