# Supplementary material for: HLA Diversity of Over 500,000 Individuals on the DATRI Register Across 18 Regions in India
Source: HLA. 2026 Sep 17;108(3):e70908. doi: 10.1111/tan.70908 (PMC13584844; doi:10.1111/tan.70908)
Supplement: Supplementary file 1 [file TAN-108-e70908-s003.docx]

Figure S1: Histogram with 100 bins of the number of individuals on the DATRI register by the relative probability of their diplotype prediction based on our EM-based algorithm for calculating haplotype frequency.

Figure S2: Number of distinct alleles by gene (A), haplotypes (B), and genotypes (C) when singletons for each of these have been removed and the remaining are grouped into how many regions they were observed in.

**C**

**B**

**A**

Figure S3: For all 18 regions, the cumulative percentage of donors who are covered for their most likely haplotypes, with haplotypes are sorted by the haplotype frequency rank (x-axis). Dotted lines and arrow indicate when 50% of a region’s population is covered by the annotated number of haplotypes.

Supplementary Data 1: ARD group designations and the alleles present in the dataset that map to the designations. Generated using IPD-IMGT/HLA Database version 3.57.

Supplementary Data 2: Allele frequencies for HLA-A, -B, -C, -DRB1 and -DQB1, 5-locus haplotype frequencies and their LD status for each of the 18 geographical regions.

Supplementary Data 3: Pairwise Hedrick’s *D’* values for overall LD between loci for each of the 18 geographical regions.

Supplementary Data 4: Pairwise Nei’s standard genetics distance between each of the 18 geographical regions based on 5-locus haplotype frequencies. Inter-region diversity and intra-region diversity metrics.
